# Positive effects of public breeding on US rice yields under future climate scenarios

**DOI:** 10.1073/pnas.2309969121

**Published:** 2024-03-18

**Authors:** Diane R. Wang, Sajad Jamshidi, Rongkui Han, Jeremy D. Edwards, Anna M. McClung, Susan R. McCouch

**Affiliations:** ^a^Department of Agronomy, Purdue University, West Lafayette, IN 47901; ^b^Department of Plant Sciences, University of California, Davis, CA 95616; ^c^Dale Bumpers National Rice Research Center, United States Department of Agriculture - Agricultural Research Service, Stuttgart, AR 72160; ^d^Section of Plant Breeding and Genetics, School of Integrative Plant Science, Cornell University, Ithaca, NY 14853

**Keywords:** genetics, historical weather, yield, machine learning, predictive modeling

## Abstract

In this study, we model and predict rice yields by integrating molecular marker variation, varietal productivity, and climate, focusing on the Southern U.S. rice-growing region. This region spans the states of Arkansas, Louisiana, Texas, Mississippi, and Missouri and accounts for 85% of total U.S. rice production. By digitizing and combining four decades of county-level variety acreage data (1970 to 2015) with varietal information from genotyping-by-sequencing data, we estimate annual historical county-level allele frequencies. These allele frequencies are used together with county-level weather and yield data to develop ten machine learning models for yield prediction. A two-layer meta-learner ensemble model that combines all ten methods is externally evaluated against observations from historical Uniform Regional Rice Nursery trials (1980 to 2018) conducted in the same states. Finally, the ensemble model is used with forecasted weather from the Coupled Model Intercomparison Project across the 110 rice-growing counties to predict production in the coming decades for Composite Variety Groups assembled based on year of release, breeding program, and several breeding trends. Results indicate positive effects over time of public breeding on rice resilience to future climates, and potential reasons are discussed.

## Significance

Rice production in the United States offers a unique model system with which to link genetics and climate change. Here, we model the relationship between genetic variation, yield, and weather using acreage data from the Southern U.S. rice-growing region from 1970 to 2015. We find evidence of positive effects of public breeding on rice resilience to future temperature and precipitation the by comparing predicted performance of groups of more recently released varieties to that of groups of older varieties. In contrast, no differences in relative yield performance were detected between groups of varieties released by state-located breeding programs. Our study provides strategies to examine the relationship between genomic variation and climate resilience that may be extended to other economically important crop species.

The resiliency of agricultural systems in response to a changing climate is of critical importance to support sustainable production of food, fiber, and feed for a rapidly expanding global population. One approach to addressing uncertainty in current crop production systems is to evaluate how weather has influenced yields historically as a means to predict future response trends. To this end, researchers have leveraged various long-term public datasets to characterize interactions between climate, management, and breeding (1–3). Several retrospective studies provide evidence that U.S. maize yields have benefited from climate trends given appropriate management adaptation over the past 30 to 40 y (4–6). Other work suggests that winter wheat in the central United States will experience overall negative effects of warming temperatures despite positive effects of reduced freezing exposure (7). For rice, there has been evidence of both shortening and prolonging phenology in rice cultivars as a result of historical weather trends (8, 9). It would be useful to link genomic variation to commercial yields in response to historical weather to predict the potential impact of breeding on crop resiliency in changing climates. However, this has been challenging due to difficulties in acquiring appropriate data that can be used to directly link across these disparate scales. Here, we demonstrate an approach to empirically model the effects of historical weather, genomic variation, and their interactions on commercial yields in the rice production system in the United States over 45 y (1970 to 2015).

Globally, *Oryza sativa* (cultivated Asian rice) is one of the most important crop species as it is the primary source of calories for the world’s poorest populations. In the United States, rice production alone generates $2.6 billion in economic output annually (10, 11). Although rice farming is typically associated with Asian agriculture, the United States consistently ranks in the top five rice-exporting nations, shipping around 45% of its production annually, mostly to Mexico, Central America, and Haiti United States Department of Agriculture, Economic Research Service (USDA ERS). Over 85% of total U.S. rice acreage is localized in the Mississippi Delta region in the states of Arkansas, Louisiana, Texas, Mississippi, and Missouri (2022 season; National Agricultural Statistics Service).

While rice has been cultivated in the United States since the 1600s using cultivar introductions, research and breeding targeted to Southern environments did not begin in earnest until the turn of the 20th century with the establishment of rice experiment stations in Louisiana (1909), Texas (1912), and Arkansas (1927) (12). Since then, breeding efforts have focused on increasing harvestable yield and grain milling quality (13), introgressing pest and disease resistance from novel genetic sources (14), and introducing herbicide tolerance traits using nontransgenic approaches (15). Given its short breeding history and localized production area, the Southern U.S. rice gene pool is relatively narrow (16, 17), originally tracing back to just several dozen founder lines (18). This, in conjunction with *O. sativa*’s self-pollinating mating system and small genome size (just 430 Mb as compared to 2.5 Gb of maize and 17 Gb of wheat genomes), facilitates the genomic characterization of Southern U.S. rice gene pools. Thus, this is an ideal model system for testing methods examining historic impacts of weather and genetics on commercial yields.

The key to leveraging genome-level variation for commercial yield prediction is knowledge of which varieties were grown historically, and at what proportions, over time and space (19). The U.S. rice community has documented this information at the county-level (hereafter referred to as variety acreage reports), albeit in records that are fragmented and not readily accessible. For example, the Rice Millers Association (RMA) tracked variety acreage for all rice-producing states until the 1980s at which time the Rice Technical Working Group picked up the effort. Individual states also surveyed this information but only in certain years. Older variety acreage data were typically available only as hard copies on file with key individuals in the rice research community.

In this study, our objectives were to 1) locate, digitize, and assemble county-level variety acreage reports across the Southern U.S. rice-growing states from 1970 to 2015 and make available the compiled data, 2) leverage this unique dataset as the critical link to develop models integrating molecular variation with historical weather data and rice production at the commercial-level, and 3) use these models to predict the impacts of past Southern U.S. rice breeding efforts on developing improved gene pools that will have long-term climate resilience. The approaches presented here can be extended to other major crop species for which for which multi-year, variety-level county acreage reports are available.

## Results

### Variability across Time and Space in Yield, Weather, and Genetics of Rice Production in the Southern United States.

**Fig. 1.**
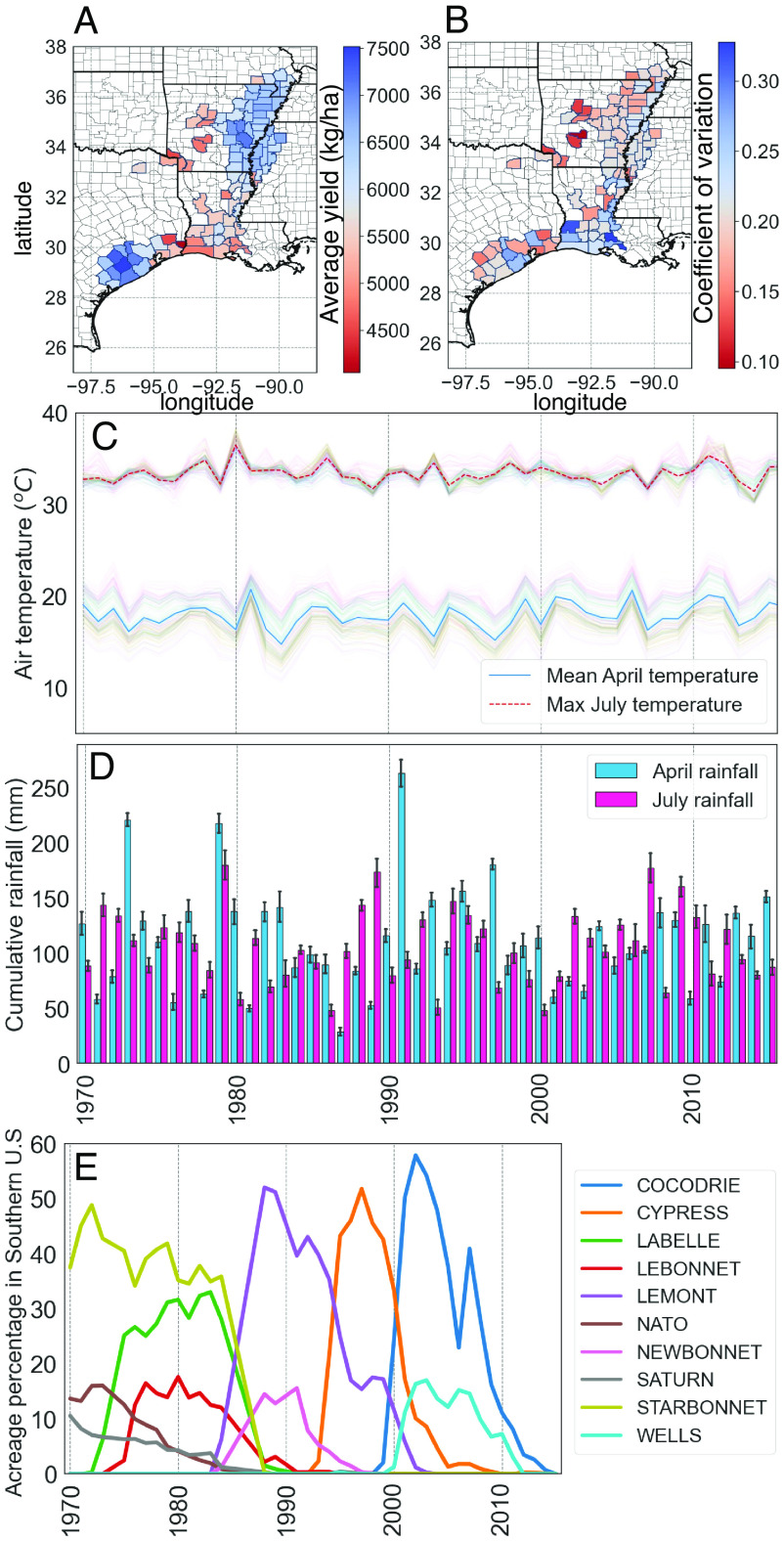
Variation in historical rice yield and weather in the Southern U.S. rice-producing region during 1970 to 2015. Average (*A*) and coefficient of variation (*B*) of yield across the 110 counties of the rice-producing region. Averages of daily mean air temperature in April (°C) and averages of daily max air temperature in July (°C) are shown in (*C*) and cumulative rainfall for April and July (mm) are shown in (*D*) for these same counties across the same time period. Translucent lines in (*C*) and error bars in (*D*) reflect variation among counties. (*E*) Change in yearly acreage over time of the ten most widely grown varieties as determined by cumulative acreage over the study period.

County-level rice yields in the Southern U.S. rice production area averaged 4035 to 7510 kg/ha during the time period of interest (1970 to 2015), while the year-to-year coefficient of variation for yield on a per county basis during this same timeframe ranged from 0.095 to 0.36 (Fig. 1 *A* and *B*). In general, Texas counties west of Galveston Bay showed the highest average yields, while counties in Louisiana had the lowest historical average yields and highest yearly variability (Fig. 1 *A* and *B*).

Previous work examining the historical impacts of weather variables on county-level rice yield in this region determined that temperature and precipitation during the months of April and July were the most important (19). These months correspond to particularly sensitive developmental stages of the rice crop: stand establishment and early canopy development (April) and flowering (July); therefore, we targeted these months for modeling, using average temperature [°C] and cumulative precipitation [mm] for April and maximum temperature [°C] and cumulative precipitation [mm] for July. During 1970 to 2015, the county-level mean April air temperature ranged from 14.7 to 20.7 °C and maximum July temperature ranged from 31.4 to 36.5 °C across the 110 rice-growing counties in this region (translucent lines) while April and July rainfall ranged 28.6 to 283 mm and 48.2 to 179.8 mm, respectively (Fig. 1 *C* and *D*). Examining historical trends, the region’s average minimum and maximum air temperatures and rainfall during the rice-growing season (April to September) have increased 0.29 °C/decade (statistically significant), increased 0.14 °C/decade (statistically not significant), and decreased by 2.2 mm/decade (statistically not significant), respectively, over this 45-y span (*SI Appendix*, Fig. S1).

The compiled variety acreage dataset contained a total of 3,738 county-year observations from 1970 to 2015. While private hybrid cultivars are grown on 25 to 50% of the acreage in the southern states today, they accounted for low proportions in our historic dataset and were unavailable for genotyping; thus, they were excluded from the model (*SI Appendix*, Fig. S2) (*Materials and Methods*). Individual inbred varieties were in production, on average, for 6.3 y across 35 counties. However, there was wide variation, and many varieties (*n* = 30) persisted on the market for over 10 y within the timeframe examined (Dataset S1 and Fig. 1*E*). The variety with the greatest persistence was cv. Saturn, which appeared in the first year of our dataset (1970) and was last grown in 2003, in total appearing in 21 different years across 40 counties during the 45-y span. On a cumulative acreage basis, the top five most widely grown varieties were cvs. Starbonnet (8.7M acres), Labelle (7.5M acres), Cypress (7.4M acres), Lemont (6.3M acres), and Cocodrie (6.2M acres). Examining trends in planting composition, greater numbers of unique varieties have been grown in recent years than in the past; for example, prior to 1990, three to five varieties (median) were grown per county while after 1990, this median value increased to five to seven varieties (*SI Appendix*, Fig. S3).

### Modeling Counties as “Bags of Alleles”.

To model the historical interactions between weather and genetics that affect yield at a field production level, we genotyped 153 varieties; this included all of the inbreds (*n* = 106) in the variety acreage dataset as well as others in the parentage of Southern U.S. rice breeding programs. Filtering the variety acreage dataset to keep only county-year observations for which 80% or more of the grown varieties were genotyped, we transformed these data into historical county-level allele frequencies across the 1970 to 2015 time period. This method considers county-year observations as “bags of alleles,” i.e., historic aggregated allele frequencies, which could be used directly to model county-level yields (*Materials and Methods* and *SI Appendix*, *Text*). A total of 2,809 total county-year records from 110 counties across the four decades were left for model development after filtering.

**Fig. 2.**
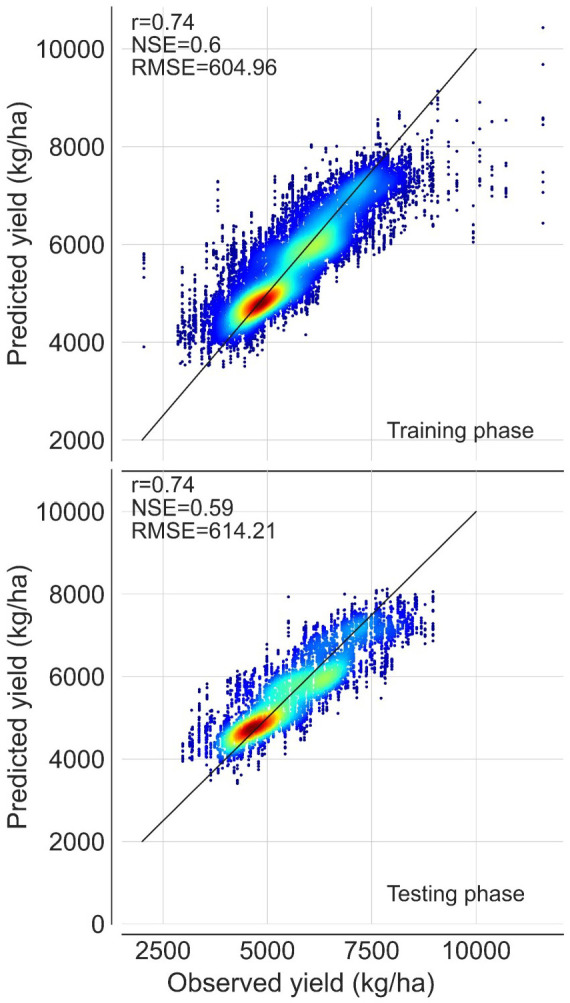
Cross validation results of the ensemble model. Results of training and testing of the ensemble model developed from ten models using a two-layer meta learner stacking approach. Color reflects number of points (red = greatest; blue = fewest) and lines indicate a 1:1 relationship. Cross-validation results from individual models are shown in *SI Appendix*, Fig. S4.

In conjunction with these county-year historical allele frequencies, our modeling framework leveraged annual county-level weather (April and July temperature and precipitation), and their interactions to model historical county-level rice yields (1970 to 2015). We selected ten machine learning models from two overarching meta-learner algorithms (regression-based and decision tree) to build an ensemble modeling framework for rice yield predictions. The final ensemble model had a Nash–Sutcliffe efficiency (NSE) score of 0.61 in the training phase and 0.60 in the testing phase and root mean squared error (RMSE) values of 604.96 and 614.21 kg/ha in the training and testing phases, respectively (Fig. 2). The ten individual models also showed acceptable accuracies (NSE: 0.44 to 0.65; r: 0.61 to 0.81; RMSE: 508.84 to 670.54 kg/ha). While the main objective was to develop an ensemble model rather than compare individual model performance, we observed that decision tree-based models showed superior performance compared to regression-based models overall (*SI Appendix*, Fig. S4).

### Model Evaluation Against Regional Trials.

**Fig. 3.**
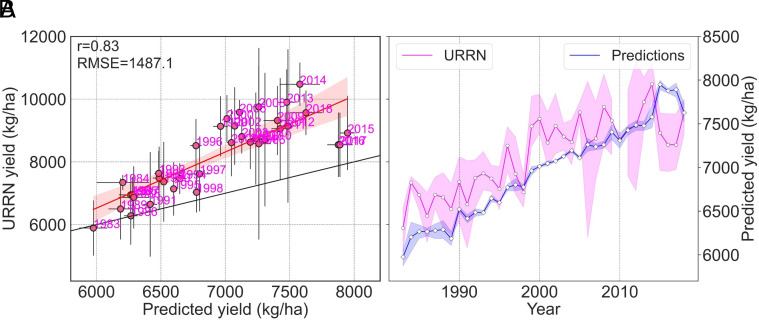
External evaluation of the ensemble model against regional yield trial data. Comparisons of rice yield predicted by the ensemble model against URRN yields across four states. (*A*) Predicted versus observed yields. Error bars reflect variation among states in each year. The black line indicates the one-to-one line. (*B*) Time series of predicted and observed yields. Shaded regions indicate SD across states. Note that in panel (*B*), observed and predicted yields utilize the left and right y-axes, respectively.

Ensemble model performance was high in the cross-validation testing phase, and to further test the validity of our predictions, we wanted to evaluate its capacity to predict yields from a completely different type of historical dataset. Through partners in the rice breeding community, we collated historical records from the Uniform Regional Rice Nursery (URRN) conducted during 1983 to 2018. The URRN is a cooperative trial carried out at one location in each of the five southern states and is used for testing adaptation of southern elite breeding lines across the rice production region. Each year, a common set of 200 entries are evaluated in a replicated trial using optimized production practices at each of the state rice breeding stations where a suite of traits, including yield, are measured. Over time, the entries change as newly developed varieties and breeding lines replace older ones. We compiled these data from Arkansas, Louisiana, Mississippi, and Texas, resulting in 3,508 yield observations directly used for model evaluation (*n* = 83 genotyped varieties, including varieties not used for training the ensemble model). In the same manner that our ensemble model was developed to predict from aggregated “bags of alleles” at a county level, we tested the model against yearly URRN composite variety groups (CVGs) (*Materials and Methods*) (*SI Appendix*, Fig. S5). The simulated yield for the URRN CVGs in each year was compared against their observed average yield as reported in the URRN records (Fig. 3).

Despite the URRN dataset being very different compared to the dataset used for developing the ensemble model (e.g., spatial scale, management differences between research trials and commercial production, weather data aggregation), we found that the ensemble model could accurately predict both the annual yields (Fig. 3*A*) and trend (Fig. 3*B*). Model evaluation in this external dataset had an *r* value of 0.83 and RSME of 1487.1 kg/ha. However, predicted yields of URRN CVGs (average of 6784.51 kg/ha; SD = 676.99 kg/ha) had a lower range in absolute values from observed trial yields (average of 8233.77 kg/ha; SD = 1195.95 kg/ha). This is not unexpected, as rice is planted in relatively small research plots with optimal inputs to maximize yield potential in the URRN trials. On the contrary, county-level yield data from NASS are aggregated across a wide range of production environments that span over a 1,200 km longitudinal distance. Therefore, this lower range in predicted yield is mainly due to the fact that the ensemble model was developed from production-level training data while the external evaluation data came from highly managed nursery trials. In spite of these marked contrasts in data sources, the ensemble model was able to predict relative differences between composite groups of varieties from the URRN trials.

### Predicted Performance of Varietal Groups Under Future Conditions.

**Fig. 4.**
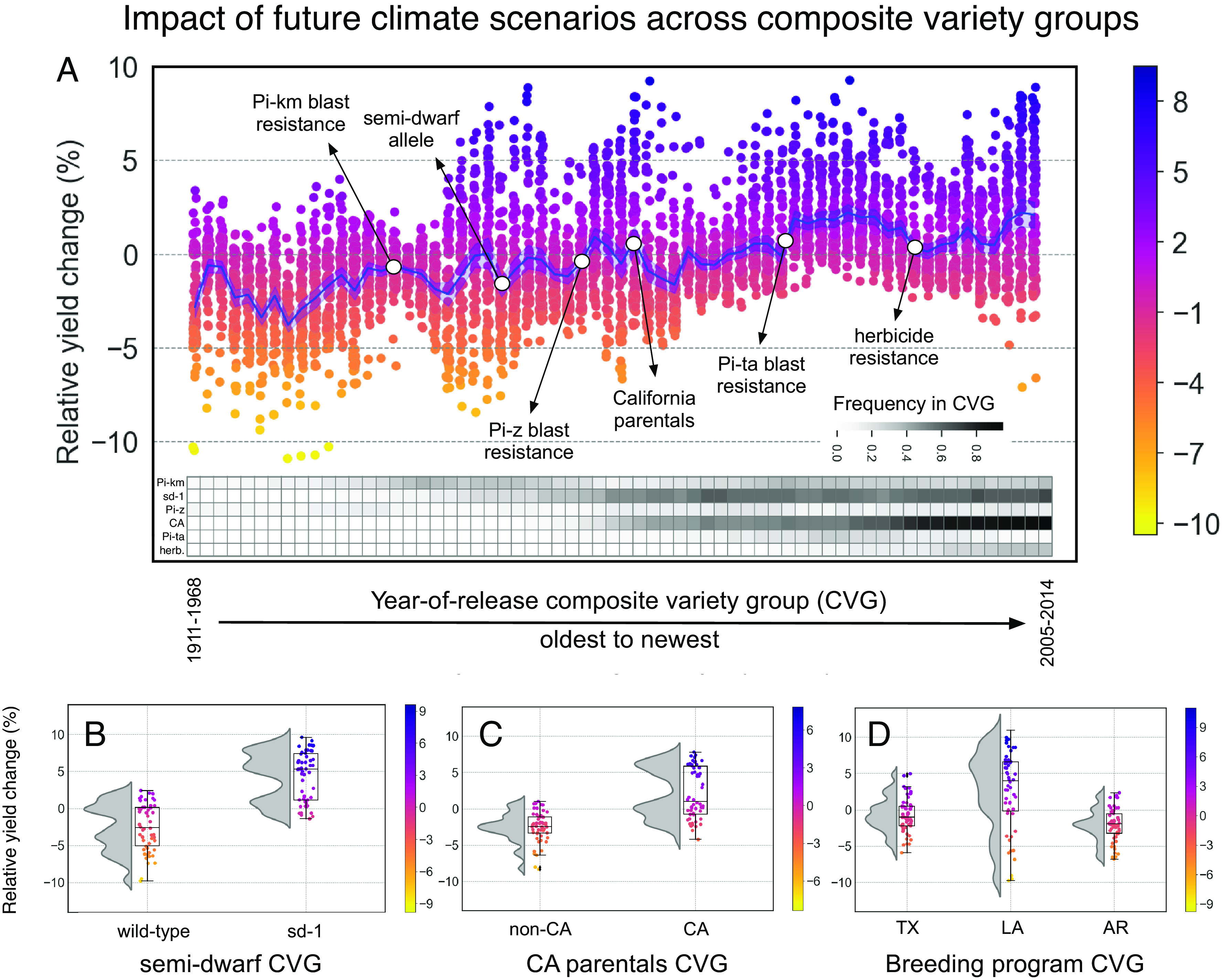
Forecasted yield performance of CVGs. Relative yield changes were computed using model-forecasted yields (2016 to 2100) relative to the average of model-backcasted yields (1970 to 2016) of the same CVG. (*A*) Comparison of CVGs classified based on their year-of-release. Groups were assembled using a sliding window approach with bins of 20 genotypes and a step size of one variety. The range of release year for each CVG shown is indicated in *SI Appendix*, Fig. S7. Red dots indicate specific breeding trends and mark the earliest CVG in which at least 20% of the CVG members have incorporated that technology. Frequency of breeding trends in each CVG is shown as a heatmap (Pi-km, Pi-z, and Pi-ta = blast resistance genes; sd-1 = semidwarf gene; CA = California parentals). (*B*) Comparison of CVGs classified based on whether they contain the semidwarf allele (sd) or not (wild). (*C*) Comparison of CVGs classified based on whether they have California rice pedigree in their genetic backgrounds (CA) or not (non_CA). (*D*) Comparison of CVGs based on their breeding program origin. TX = Texas; AR = Arkansas; and LA = Louisiana.

The cross- and externally validated ensemble model was next used to project yields for the southern U.S. rice genepool under future climate conditions. To address the question of how public breeding over time will likely impact potential future performance, a sliding window approach was taken to compare CVGs using varieties from the historical variety acreage dataset (*Materials and Methods*). Each of these CVGs was composed of 20 varieties defined based on their year of release, between 1911 and 2015, with the objective of comparing the performance of older versus newer varieties. In all cases, forecasted performance was compared to the same CVG’s own “backcasted” historical performance to obtain a relativized metric as the basis of comparison across CVGs (*Materials and Methods*). Overall, CVGs comprised of varieties released in 1995 and prior were predicted to be detrimentally affected by future climate conditions as compared to historical conditions (CVG average values for the first and third quartiles of −2.0 to −0.5% relative yield change, respectively). Contrastingly, CVGs comprised of varieties released in 1996 and after were forecasted to experience a slight benefit under future conditions (CVG average values for the first and third quartiles of 0.35 to 1.8% relative yield change under future conditions). Taken together, results of the ensemble modeling indicated positive effects of recent public breeding efforts on overall resilience to future climate with respect to April and July temperature and precipitation (Fig. 4*A* and *SI Appendix*, Figs. S6 and S7).

There have been several notable breeding trends during the time frame of the historical modeling period. These include the introduction of pest and disease resistance (e.g. rice blast resistance genes *Pi-km*, *Pi-z*, *Pi-ta*), herbicide resistance (Clearfield technology), short stature semidwarf allele [*sd-1*], early vigor and altered seed size and quality (via crosses with California germplasm) (Fig. 4*A*). To determine whether there was a potential impact of these breeding options on forecasted performance, we compared CVGs classified based on these features (Fig. 4 *B* and *C*) and found that those carrying the semidwarf allele and/or California ancestry showed a more positive predicted yield change compared to CVGs that did not.

Finally, we investigated the predicted performance of CVGs classified based on breeding program. Overall, means of distributions were not significantly different in forecasted yields between the three breeding programs Arkansas [*n* = 25], Louisiana [*n* = 34], and Texas [*n* = 32] (Fig. 4*D*). However, the Louisiana CVG had greater variance in forecasted yields than either of the other two states. Examining backcasted yields only, all three breeding program CVGs showed a similar range between 6165 and 6445 kg/ha.

## Discussion

In this study, we modeled historical rice yields and predicted future performance by explicitly accounting for genetics and their interactions with weather. Our results indicate positive effects from recent public breeding efforts in the Southern United States where the current germplasm base demonstrates the potential for climate resilience, with older varieties more susceptible to detrimental effects under future climates based on temperature and precipitation relative to more modern varieties. This is consistent with a recent study that examined five decades of continuous cropping of irrigated rice in Asia where new varieties helped to offset the negative effects of changing climate (3). However, that study also suggested that the pace of genetic improvements was not adequate to meet the projected demand for global rice production. On the other hand, our results contrast with empirical modeling work in U.S. winter wheat using historical variety trials, where more recently released varieties which have a longer grainfill phase were less tolerant to heat stress during this period than older varieties (7). Direct comparison of our results with previous reports of forecasted relative yield changes in U.S. rice is challenging due to the fact that the varieties used for model training/calibration are not typically reported.

Upon examining county-level yields, we observed regional differences among the Southern U.S. rice-growing states. Arkansas has the greatest rice acreage in the United States along with relatively high average yields and low variability. This may be due to soils which are amenable to crop rotation along with relatively low disease pressure and shorter periods of extreme heat. Conditions and productivity are similar in the more limited rice-growing area in Mississippi. Toward the south, Louisiana ranks second in acreage, but is marked by lower and more variable yields due to extensive periods of heat, high humidity which increases disease pressure, a soil hardpan which prevents crop rotation, and management practices that accommodate rotation with crawfish production. Although climatic conditions are similar to Louisiana’s, the rice acreage in Texas is about half that of Louisiana and primarily located on the lower Gulf Coast where soils are amenable to crop rotation, the disease pressure is lower, and, unlike Arkansas, a high percentage of the acreage is ratoon cropped adding to total yields (which may explain higher average nitrogen rates; *SI Appendix*, Fig. S8). While not accounted for in our modeling efforts here, if these soil types and management differences were explicitly represented in our ensemble model, we might expect improvements in prediction.

During the modeling timeframe, Southern U.S. rice was impacted by several notable breeding trends. These include the deployment of blast resistance genes *Pi-km* (1934) and *Pi-ta* (1990) along with the development of semidwarf varieties (starting in the 1980s) all of which introduced genomic variation from the indica subpopulation. In each case, the trait of interest originated from a limited number of *indica* donors; selected backcross-derived offspring were used repeatedly in U.S. variety development in the following decades as is evident from pedigree information USDA-ARS Germplasm Resources Information Network (GRIN). Simultaneously, incorporation of more *temperate japonica* alleles from California germplasm and, more recently (2002), wide deployment of a mutation from a U.S. *tropical japonica* variety that confers herbicide resistance (20), also impacted the gene pool. Due to these and other breeding trends that overlap in time, it is difficult to pinpoint if these are directly responsible for the beneficial trajectories observed when forecasting year-of-release CVGs (Fig. 4*A*), as they each demonstrate newer CVGs outperforming older ones (e.g., Fig. 4*C* and *D*). However, our results suggest that past efforts to incorporate either single major genes from *indica* donors or specific traits from *temperate-tropical* admixture donors, may have also inadvertently introduced additional genomic variation that has brought greater adaptability and climate resilience. It is challenging to identify exactly which genomic regions are responsible for significant interactions with weather due to the fact that county-level allele frequency patterns represent groups of SNPs that are localized across the genome rather than in single regions (*SI Appendix*, Fig. S9). This implies that base broadening breeding efforts, invested in by public institutions, may result in opportunities to address future unidentified challenges. Finally, classifying rice varieties by their source breeding program (i.e., Louisiana, Arkansas, and Texas), we found that CVGs performed similarly, on average, under forecasted future climates and speculate that this may be a consequence of a sustained narrow gene pool, historical germplasm sharing across these public breeding programs, as well as having similar growing environments and breeding targets across this relatively small area in the southern states. However, the Louisiana Composite Variety Group was shown to have greater forecasted variability, and one potential explanation is that this CVG was marked by more extensive use of *temperate japonica* California varieties as parentals and a higher proportion of medium grain releases (13 in LA, versus six in AR and three in TX), which are admixtures of *temperate japonica* and *tropical japonica* germplasm.

Acknowledging that crop yield is also greatly influenced by management and their interaction with genetics over time, we checked whether overall modeling conclusions remained valid regarding the performance of CVGs with the inclusion of nitrogen fertilizer as a proxy for management trends (*SI Appendix*, *Text*). There were differences in individual CVG predictions compared to our original analysis, and overall predictions of relative response of CVGs to future climate scenarios saw a downward shift (*SI Appendix*, Fig. S8). However, the observation that CVGs composed of more modern varieties showed greater relative resilience under future scenarios compared to CVGs composed of older varieties remained valid. For example, CVGs assembled only by varieties released 1996 and after had, on average, 2% greater relative yield response under future scenarios than CVGs composed only of varieties released 1995 and prior. This was true whether N was considered explicitly in the models or not.

The current study applies empirical modeling approaches to historical datasets and utilizes an overall ensemble model for prediction of forecasted and backcasted yields. Like all empirical methods, this framework can have high accuracies of prediction for scenarios similar to those found in the training dataset yet should be interpreted with great caution under scenarios that contrast markedly with those in the training dataset (21). While historical records in precipitation and April temperature reliably capture the range predicted under future conditions, future warming predictions for maximum July temperature fall over 4 °C beyond the range observed in the four decades of historical records (*SI Appendix*, Fig. S10). To account for this, we did not keep scenarios that had forecasted maximum July temperatures (coinciding with rice anthesis) above 40 °C; this threshold was informed by previous physiological studies (*Materials and Methods*) and resulted in dropping 36% of scenarios for SSP5-8.5, most of which occurred after 2068, and dropping just 5% for the SSP1-2.6 (*SI Appendix*, Fig. S6). Thus, we limited our analyses to results from SSP1-2.6 (Fig. 4). Notably, when we investigated the ensemble model response to single weather variables (*SI Appendix*, *Text*), results were consistent with current understanding of rice physiology (*SI Appendix*, Fig. S10), and older CVGs demonstrated more variability compared to more modern CVGs (*SI Appendix*, Fig. S11). Temperatures above 40 °C would likely trigger a change in land use, and predicting such changes should be part of follow-up analyses.

The frequency of extreme weather events, including storm surges, hurricanes, and associated disease pressures, is predicted to increase in the future. These events are not accounted for in an empirical modeling framework such as the one presented here. Part of the Southern U.S. rice-growing region is located in a particularly storm-prone area along the Gulf Coast, and extreme weather-associated events contributed to low performance in one or more locations of URRN trials in 2011, 2012, 2015, 2016, and 2017 (e.g., Hurricanes Isaac and Harvey) (Fig. 3*A*). While breeding would be ineffective in the face of extreme events such as hurricanes, breeders may want to consider specific targets for tolerance to high temperature stress at anthesis in addition to selection for improved lodging resistance as well as overall yield and grain quality. These kinds of efforts are also relevant for hybrid rice, which were not modeled here due to data limitations, but are increasing in acreage and are also very susceptible to high temperature stress during flowering (22), with sensitivities depending on the parentage (23). While it is challenging to draw general conclusions about differences in heat tolerance between inbred and hybrid rice due to the strong impact of the specific genetics of each genotype, one study reported higher heat susceptibility in the hybrids tested compared with inbred varieties (24). Strategies for improving heat tolerance in both inbreds and hybrids could include introducing resilient donors from exotic material (e.g., those that have useful traits such as early time-of-day of anthesis) as well as training new genomic prediction models under heat stress environments, and identifying specific genes and genomic variants (25) that could be targeted to improve heat tolerance in *tropical japonica* rice. Management adaptations are also an option, such as shifting to earlier planting dates to avoid high temperature during anthesis and employing ratoon cropping (26–28). In addition to genetic and cultural adaptations, land use patterns may need to change to sustain food production (29).

An additional limitation of empirical studies that use historical datasets for future crop production prediction is the inability to account for CO_2_ trends. Based on the historical past and forecasted future, atmospheric CO_2_ levels will continue to rise. Considering limitations already described for empirical models to predict beyond a training set range, we could not incorporate CO_2_ effects into our model given that future levels are predicted to be well beyond the range experienced historically. Moreover, the interaction of increasing CO_2_ and temperatures is expected to impact yields in a nonlinear manner (30). In light of these complexities, our historical datasets, which we have made publicly available, can be used in process-based crop modeling frameworks such as those proposed by Messina et al, which offer a formal integration of genomics and process-based models (31). These models, in contrast with empirical approaches, are developed based on physiological parameters and can directly account for feedback and feedforward behaviors inherent in complex systems, such as the effect of rising CO_2_ on stomatal closure which can lead to photosynthetic acclimation and decreased capacity for transpirational cooling. When parameterized with knowledge about genotype (varieties), either using physiological or genomic information, these kinds of models can be used to predict which genotypes may outperform others and under which specific climatic scenarios (32, 33). Bridging information from genome variation to landscape-level yields is key to evaluating the resiliency of rice production under increasing climate uncertainty. Our study provides strategies to examine these linkages across scales and may be extended to other economically important species to further examine crop resiliency in the face of changing climates.

## Materials and Methods

### County-Level Variety Acreage Data.

Information indicating county-by-year acreage planted for each variety between 1970 and 2015 in the Southern rice-producing states was obtained online and directly from rice researchers at the Louisiana State University Agricultural Center, Mississippi State University, Texas A&M AgriLife Research and Extension Center, and the Arkansas Rice Research and Extension Center and assembled into a single dataset. Older records, for which only hard copies were available, were obtained in 2014, scanned, and manually digitized at Cornell University.

### County-Level Allele Frequency (“Bag of Alleles”).

Seeds from 153 rice accessions were obtained from Dale Bumpers National Rice Research Center and planted at the Guterman Bioclimatic Facilities at Cornell University (Ithaca, NY, USA). Tissue from resulting seedlings was collected and genotyped using Genotyping-By-Sequencing (34); *SI Appendix*, *Text*. Genotyped varieties included those from the variety acreage dataset, U.S. rice progenitors (landraces), parents used in Southern U.S. breeding crosses over the last century, and new varieties released after the time period used for modeling (*SI Appendix*, Fig. S13). The variety acreage dataset was filtered first to keep only county-year observations where at least 80% of varieties grown were genotyped (*n* = 2,809). Next, using acreage proportions of each variety from the variety acreage dataset and binary allele calls of each variety from the genotyping dataset, aggregated county-level allele frequencies (“bags of alleles”) were computed for each year across 1970 to 2015 following

$$\begin{array}{c} c_{j}=\sum_{i}A_{i}m_{\mathrm{ij}}, \end{array}$$

where $c_{j}$ is the county-level allele frequency of marker *j*, $A_{i}$ is the acreage proportion of each variety *i*, and $m_{i}j$ is the binary allele call at marker *j* for variety *i*.

### Yield Data.

Historical county-level rice yield data were obtained from the National Agricultural Statistical Services (NASS) and at the plot level from the URRN trials (*SI Appendix*, *Text*).

### Weather Data.

For county-level modeling, gridded historical monthly weather data were obtained from PRISM (https://prism.oregonstate.edu/historical/) (35) with a spatial resolution of 4 km covering 1970 to 2015. Weather data from research stations associated with the URRN trials were obtained through the iAIMS data center (https://beaumont.tamu.edu/climaticdata) (36). Future climate data were retrieved from the Inter-Sectoral Impact Model Intercomparison Project ISIMIP, (37). *SI Appendix*, *Text* contains methods on weather data processing.

### Development of the Ensemble Model.

Ten machine learning models (CatBoost, GradientBoost, RandomForest, AdaBoost, XGBoost, LASSO, Elastic net, Bayesian Ridge, Support vector, and Stochastic Gradient Descent) were utilized to develop a two-layer meta learner ensemble model for rice yield prediction (*SI Appendix*, Fig. S11). The specific models were selected to represent a variety of learning algorithms (e.g., boosting, bagging, regularized regressions) to help reduce uncertainty of the ensemble approach. Full details are provided in *SI Appendix*, *Text* and hyperparameter values are found in Dataset S2. Briefly, county-level allele frequencies along with county-level yield and weather information and their interactions were used as inputs to model the impacts of climate on rice yield distributions, explicitly taking into account genetic information. The general model was defined in the following form,

$$\begin{array}{c} y=f(W,G,W\times G|\theta ), \end{array}$$

where *y* is yield, *W* represents weather variables (i.e., air temperature and rainfall), and *G* represents genetic parameters (i.e., county-level allele frequencies), *W*×*G* represents the interactions between weather and genetic variables, and is the impact of climate change. To define function (*f*), we used two overarching sets of ML algorithms based on regression analysis and decision trees.

The resulting training dataset based on the allele frequency, weather data, and their interactions resulted in a high dimensional dataset, where the number of features (43,779) far exceeded the number of observations (2,809). We applied the principal component analysis (PCA) to reduce dimensionality of the dataset and generated a training dataset with 85 uncorrelated principal components. The optimal number of components (i.e., 85) was determined by taking different numbers of components from 1 to 300 and evaluating the model’s accuracy during the model development phase. The number of components that resulted in the highest accuracy score was selected as optimal (*SI Appendix*, Fig. S14). During the model development, cross-validation was carried out using 75% of the data for development and tested with the remaining 25% of the data.

### External Evaluation of the Ensemble Model.

The final ensemble model was further evaluated using the URRN dataset (*SI Appendix*, Fig. S5). In this step, the varieties that had been genotyped and were in the URRN were used to inform overall allele frequencies for a yearly CVG like the “bags of alleles” concept. For as long as the variety was tested in the URRN, its genetic information contributed to the overall allele frequency, weighted by the total number of CVG varieties in the URRN each year. The CVG allele frequencies and the local breeding station weather data during 1983 to 2018 were then used as inputs into the ensemble model to generate yield predictions. These predictions were evaluated against observed average URRN yields of the varieties that constituted the CVGs.

### Backcasting and Forecasting Yields with the Ensemble Model.

Finally, the ensemble model was used to backcast and forecast yield for other CVGs using varieties found in the historical variety acreage dataset grouped based either on their year of release, their breeding program of origin, or by breeding trend (*SI Appendix*, Fig. S12). As with the URRN external evaluation process, allele frequencies of these CVGs were constructed using the binary genotype data, with equal proportions allotted to each variety in the group. Performance of these CVGs was predicted using the ensemble model along with historical weather (1970 to 2015) and future weather (2015 to 2100) for backcasting and forecasting, respectively. Note that the CMIP6 climate projections (for scenarios SSP1-126 and SSP5-585; *SI Appendix*, *Text*) were projected from the year 2015 to 2100; therefore, we considered this period for the future climate assessments. Due to nonlinear effects of very high anthesis temperature on rice yields, we considered only forecasted scenarios that did not exceed a maximum monthly July temperature of 40 °C based on previous rice physiology studies (38, 39).

## Supplementary Material

Appendix 01 (PDF)

Dataset S01 (XLSX)

Dataset S02 (XLSX)

## Data Availability

Original sources for publicly available data are described in the *Materials and Methods*. Specific subsets of public data used in this study as well as newly generated datasets and analysis scripts are available at Zenodo (https://doi.org/10.5281/zenodo.8040082) (40). These include data inputs for modeling (historical and future weather, historical county-level yields, genetic marker data, and compiled variety acreage dataset) along with three models (PCA and two meta-machine learning models), and three relevant scripts to facilitate the creation of training data and the machine learning models. Variety acreage data, rice genotype (GBS) data have been deposited in Zenodo (https://doi.org/10.5281/zenodo.8040082) (40). URRN data used for external model evaluation are available only to URRN cooperators and cannot be shared at this time. Previously published data were used for this work (Historical weather: https://prism.oregonstate.edu/historical/ (35); URRN on-farm weather: https://beaumont.tamu.edu/climaticdata (36). Future weather from CMIP6: https://esgf-node.llnl.gov/projects/cmip6/ (41). County-level rice yield data: https://www.nass.usda.gov/) (42).
