# Supplementary material for: Positive effects of public breeding on US rice yields under future climate scenarios
Source: Proc Natl Acad Sci U S A. 2024 Mar 18;121(13):e2309969121. doi: 10.1073/pnas.2309969121 (PMC10990131; doi:10.1073/pnas.2309969121)
Supplement: Supplementary file 1 — Appendix 01 (PDF) [file pnas.2309969121.sapp.pdf]

Supporting file for:

Positive effects of public breeding on U.S. rice yields under future climate scenarios

Diane R. Wang, Sajad Jamshidi, Rongkui Han, Jeremy D. Edwards, Anna McClung, Susan R. McCouch

This PDF file includes:

Supporting text
Figures S1 - S15

Tables S1-S2 are found in a separate excel sheet

Supporting Text. Extended Methods

URRN data. The Uniform Regional Rice Nursery (URRN) is a collaborative project conducted by the public rice breeding programs located in Beaumont, Texas, Crowley, Louisiana, Stuttgart, Arkansas, and Stoneville, Mississippi. Each year it includes the same 200 rice varieties and breeding lines at all locations which are evaluated using a randomized complete block design. Varieties change over time as new breeding materials are tested and others are eliminated. Yield is determined from replicated plots that are approximately 3 m^2. Local recommended cultural management practices are used to optimize productivity. Variety means at each location-year were used in the analysis.

Genotype data generation. A 96-sample multiplex Genotyping-By-Sequencing (GBS) approach (1) was used to generate Single Nucleotide Polymorphism data on 153 accessions (hereafter referred to as the Full Set). Leaf tissue samples were harvested from a single plant per accession and DNA was isolated using Qiagen DNeasy Plant Mini Kits. DNA samples were digested using *ApeK1*. Resultant raw fastq files containing the 153 samples were processed along with previously generated fastq files on 54 diverse accessions that served as population controls across all five *O. sativa* subpopulations (*temperate japonica, tropical japonica, aromatic, indica,* and *aus*). SNP data were generated using the GBS bioinformatics pipeline implemented in TASSEL3.0. Bowtie2 was utilized to align reads to the MSUv7 genome assembly. Within the combined group of the Full Set and subpopulation controls, there were 111,500 polymorphic sites. The dataset was filtered for a minimum call rate of 0.8 and a minor allele frequency of 0.01, resulting in a 8755-SNP dataset for downstream modeling.

Population structure. Population structure analysis of the Full Set (n=153) was performed with fastSTRUCTURE (1) using 111,500 polymorphic sites and Principal Components Analysis using 4704 polymorphic sites (90% call rate; 5% minor allele frequency). A phylogenetic tree was constructed using the Neighbor-Joining method implemented in Geneious 10.0.9 using 9004 polymorphic sites (90% call rate; no minor allele frequency filter).

Weather data processing. The weather data used in our modeling scheme included air temperature and rainfall during April and July from 1970 to 2015 (for historical analysis) and

2015 to 2100 (for future projections). Gridded historical monthly weather data was retrieved from PRISM (accessible from https://prism.oregonstate.edu/historical/) with a spatial resolution of 4 km covering 1970 to 2015. These monthly data were aggregated at a county level (over the study region) and were used as input to the machine learning algorithms in the model development phase. We also leveraged the historical weather dataset to gain insights into the historical variation in temperature and precipitation within the rice-growing counties. The Man-Kendall trend test was employed, and trends with a significance level of $P < 0.05$ were considered statistically significant.

Another set of historical weather data was obtained from the weather stations at the five locations of URRN field trials (from 1983 to 2018). These points-based data were accessed through the iAIMS data center (https://beaumont.tamu.edu/climaticdata) and used in the model evaluation phase. Future climate projections data were retrieved from the Inter-Sectoral Impact Model Intercomparison Project (ISIMIP, (2)). ISIMIP provides a bias-corrected and downscaled version of the CMIP6 products (the Coupled Model Intercomparison Project, sixth report, https://esgf-node.llnl.gov/projects/cmip6/). For this study, future air temperature and rainfall during April and July were obtained under two "Shared Socioeconomic Pathways" (SSPs) scenarios: SSP1-126 and SSP5-585. SSP1-126 represents a sustainable future where CO_2 emissions are cut to net zero around 2050, while SSP5-585 represents a climate without fossil-fuel restrictions where CO_2 emissions reach roughly double the current level by 2050. Future climate projections were retrieved at a daily scale with 0.5-degree spatial resolution and were re-aggregated to monthly and county levels to be used with the ensemble model for yield projections. To account for the uncertainties in future climate projections, three GCMs (General Circulation Models) were used. These were GFDL-ESM4, UKESM1-0-11, and MPI-ESM1-2HR.

Developing input dataset. The input dataset utilized in the development of our machine learning models consisted of monthly weather data, *i.e.* mean temperature and precipitation in April and maximum temperature and precipitation in July. Additionally, genetic information was incorporated, specifically county-level allele frequencies derived from 8755 alleles, along with the interactions between weather and genetic factors. The integration of these data sources

resulted in a high-dimensional dataset with notable correlations among its features. To address this issue, we employed Principal Component Analysis (PCA) as a dimensionality reduction technique. By applying PCA, we transformed the initial dataset into a lower-dimensional representation while preserving as much relevant information as possible (**Figure S11**). The resulting dataset consisted of 85 features and encompassed a total of 2809 observations. This reduced dataset was subsequently used for both the training and testing phases of our study.

Machine learning ensemble modeling approach. Ten machine learning models based on two overarching learning algorithms (regression and decision tree) were employed to develop an ensemble modeling framework. Decision tree-based models were CatBoost, GradientBoost, RandomForest, AdaBoost, and XGBoost. Regression-based models were LASSO, Elastic net, Bayesian Ridge, Support vector, and Stochastic Gradient Descent. We first trained and tested each individual model using the county-allele dataset to evaluate their overall performance and stability for our modeling framework. The models were tuned and trained based on the 75% of the dataset, and tested on the remaining 25% in 5-fold cross validation process. The hypertuned parameters were different for each of the models and their optimal values are provided in the **Table S2**. The models' performance were assessed using the Pearson correlation (r), Root Mean Squared Error (RMSE), and Nash–Sutcliffe Efficiency (NSE) using the following equations.

$$\boldsymbol{r} = \frac{\sum_{i=1}^{N}(\boldsymbol{O}_i - \bar{\boldsymbol{O}})(\boldsymbol{P}_i - \bar{\boldsymbol{P}})}{\sqrt{\sum_{i=1}^{N}(\boldsymbol{O}_i - \bar{\boldsymbol{O}})\sum(\boldsymbol{P}_i - \bar{\boldsymbol{P}})}}$$

$$\boldsymbol{RSME} = \sqrt{\frac{\sum_{i=1}^{N}(\boldsymbol{P}_i - \boldsymbol{O}_i)^2}{\boldsymbol{N}}}$$

$$\boldsymbol{NSE} = \boldsymbol{1} - \frac{\sum_{i=1}^{N}(\boldsymbol{O}_i - \boldsymbol{P}_i)^2}{\sum_{i=1}^{N}(\boldsymbol{O}_i - \bar{\boldsymbol{O}})^2}$$

where P and O represent the predictions and observations and P and O represent the mean of predictions and observations, respectively. Once the models' accuracy was assured, they were used in a two layer meta learner stacking approach to be used for final simulations. More specifically, a decision-tree based learner (*i.e.*, the XGBoost algorithm) and a regression-based learner (*i.e.*, the LASSO algorithm) were trained on the ten model predictions and the average of their outputs were considered as the final simulations. The stacking approach has been reported to have more accuracy compared to using the individual models or using the models' average

(3). The overall framework of the modeling is presented in **Figure S10**. Model development was carried out in the Python environment using the sklearn, xgboost, and catboost libraries.

Simpler models excluding explicit genetics. Simpler models omitting genetic variables were constructed to compare testing metrics. Two types of simpler models were compared, both of which included the same weather variables described above and either included Year (a) or excluded Year (b). Model (a) had better testing performance than model (b) (r = 0.66 versus b = 0.34), as Year theoretically represents technological trends over time, offering a proxy for breeding and management. Overall, performance metrics of models excluding genetic variables were not as good as the ensemble model that included genetic variables that is shown in Figure 2.

Extended model including management. Crop yield is influenced by a combination of weather conditions, genetics, and agronomic practices. Constructing a model that accurately captures this complexity necessitates reliable historical data to ensure its efficacy. While we acknowledge the influence of many other variables on yield variability, the availability of comprehensive data for all these variables is often limited. This study focuses on the genetic aspects of rice in response to the environmental variables, we additionally tested if including the management factors in our analysis would affect the outcome. Here, management was proxied by nitrogen fertilizer because of its known substantial impact on crop yield and its change in application rate over time. Thus, the rice yield was considered as a function of weather, genetics, nitrogen application, and their interactions. County-level nitrogen fertilizer data for rice were sourced from Cao et al. (2018), handling missing values and verifying data integrity. The average nitrogen application rate and its yearly variability during 1970 to 2015 are shown in **Figure S8**. Implementing this new dataset within our existing framework, we trained, hyper-tuned, validated, backcasted, and forecasted CVGs. For backcasting and forecasting, we maintained a constant county-wise nitrogen application rate, aligning with the historical average for each county.

CVG response to single weather variable. To distinguish the individual impacts of different weather variables on CVGs backcasting and forecasting was carried out for year-of-release CVGs by manipulating one weather variable at a time while holding the others constant. The constants were set based on the historical county-specific average values. For example, we recorded the response of each CVG to variations in April temperature, while keeping July

temperature, April precipitation, and July precipitation constant at their respective historical averages for each county. We followed a similar approach by altering July temperature, April precipitation, and July precipitation, while keeping the remaining variables constant.

Supporting Text. References

1. R. J. Elshire, *et al.*, A robust, simple genotyping-by-sequencing (GBS) approach for high diversity species. *PloS One* **6**, e19379 (2011).
2. L. Warszawski, *et al.*, The inter-sectoral impact model intercomparison project (ISI–MIP): project framework. *Proc. Natl. Acad. Sci.* **111**, 3228–3232 (2014).
3. Y. Ren, L. Zhang, P. N. Suganthan, Ensemble classification and regression-recent developments, applications and future directions. *IEEE Comput. Intell. Mag.* **11**, 41–53 (2016).

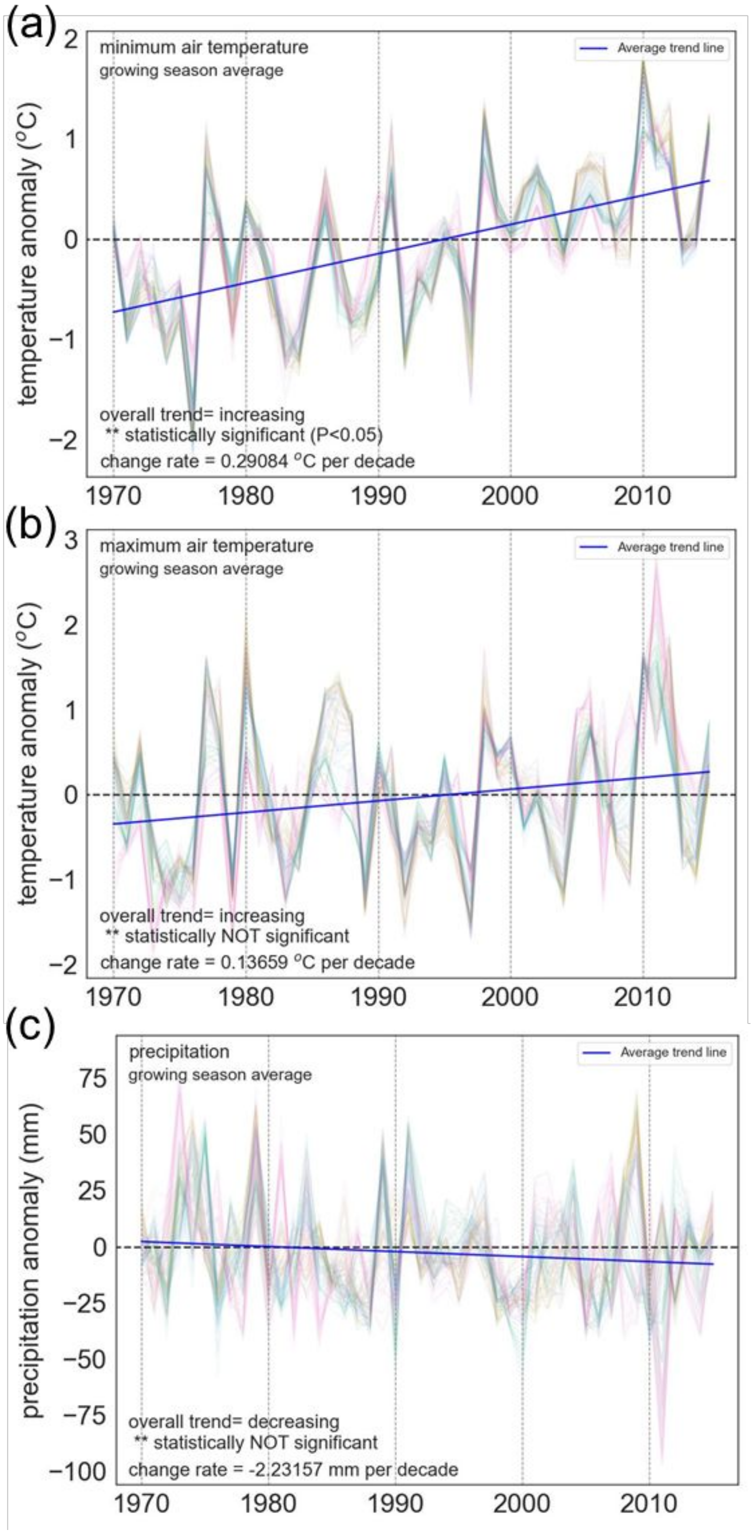

Figure S1. Trends in past weather across the study region for the April-September growing season during the historical modeling period. Trends for minimum temperature (a), maximum air temperature (b) and precipitation (c) were assessed in the southern U.S. rice cultivation areas using the Mankendal trend test during 1970 to 2015.

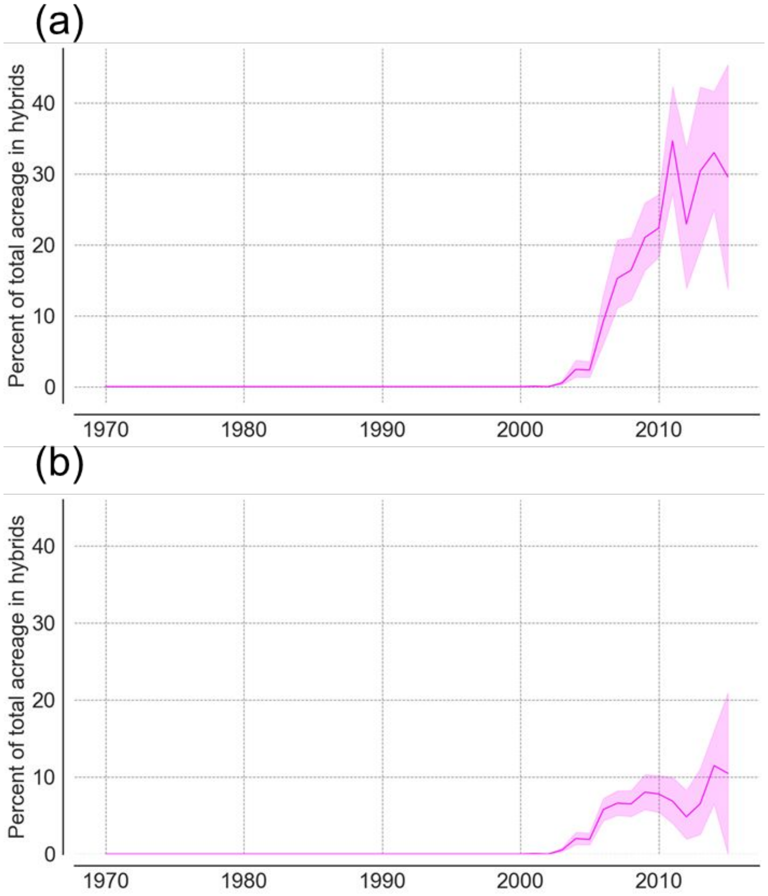

Figure S2. Acreage planted in hybrids over time. Years 1970-2015 are shown. Panel **(a)** shows the actual percentage of acreage planted in hybrids over time from the original variety acreage dataset. Panel **(b)** shows the hybrid percentage acreage after filtering to keep only county-year observations for which 80% or more of the grown varieties were genotyped. The magenta-shaded area shows the standard deviation of the planted acreage across different counties.

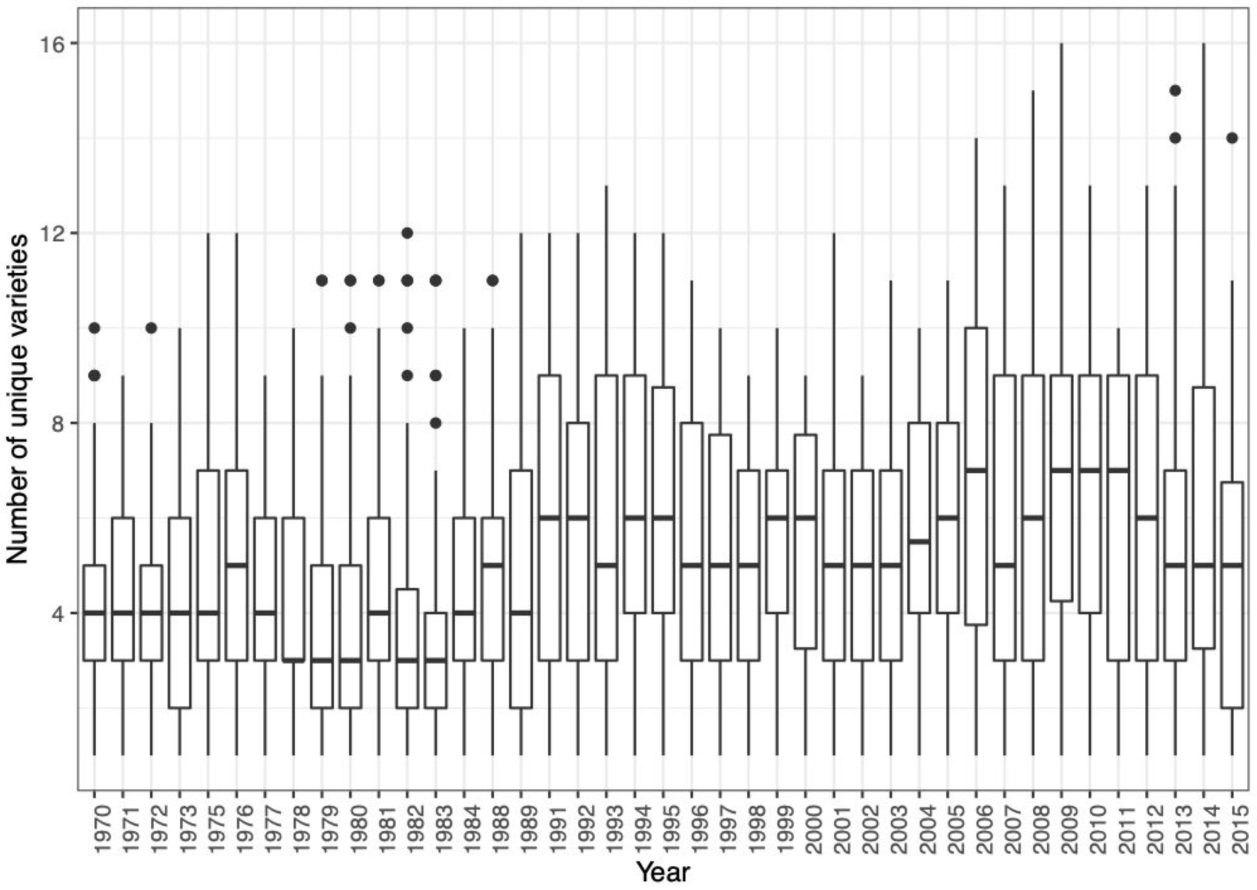

Figure S3. Number of rice varieties planted in Southern U.S. counties over time. Variation within a year reflects differences among counties.

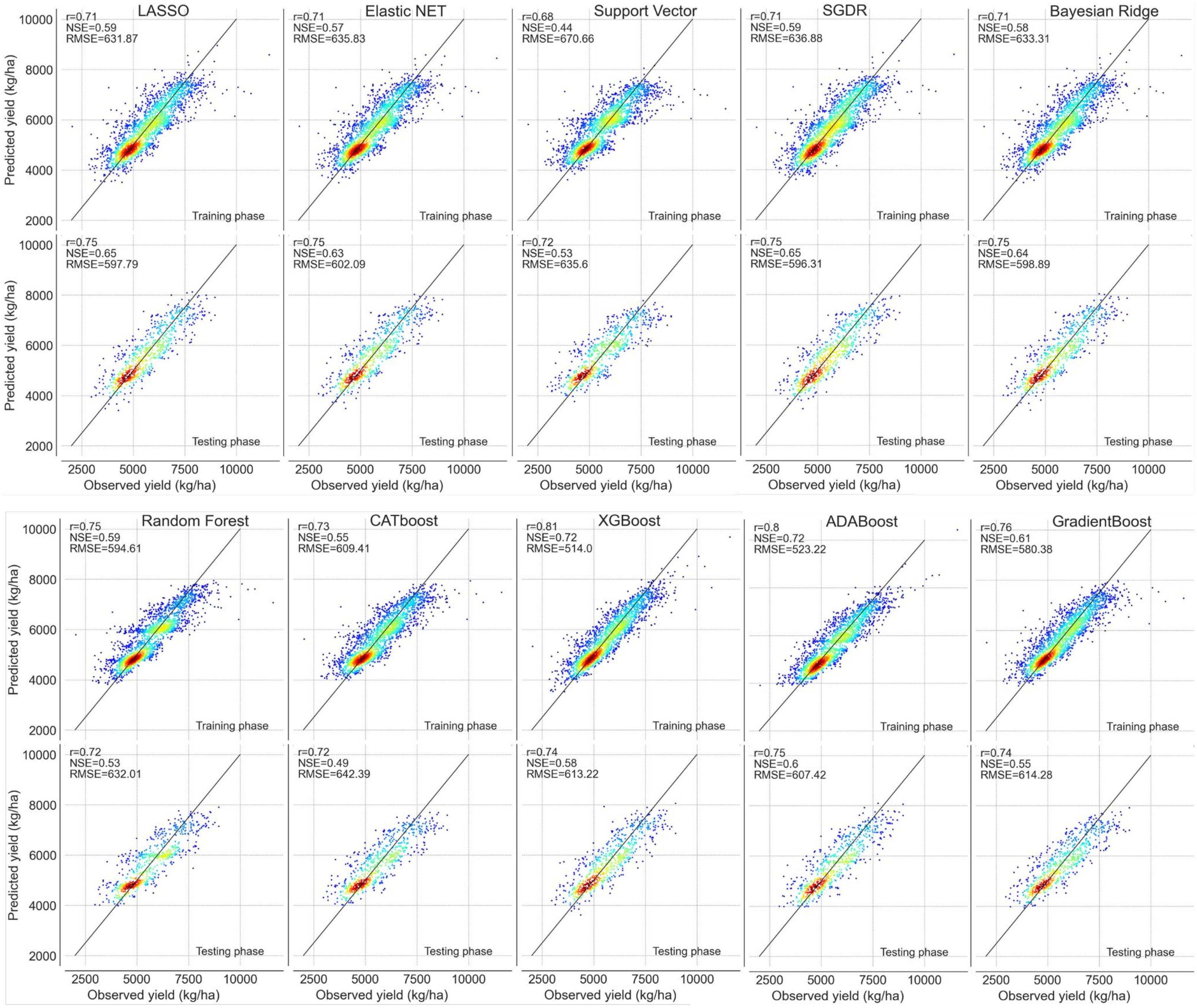

Figure S4. Single model performance during the training and testing phases. Training and testing were performed by dividing the county level allele frequency and yield data at 75% and 25%, respectively. Color reflects number of points (red = greatest; blue = fewest).

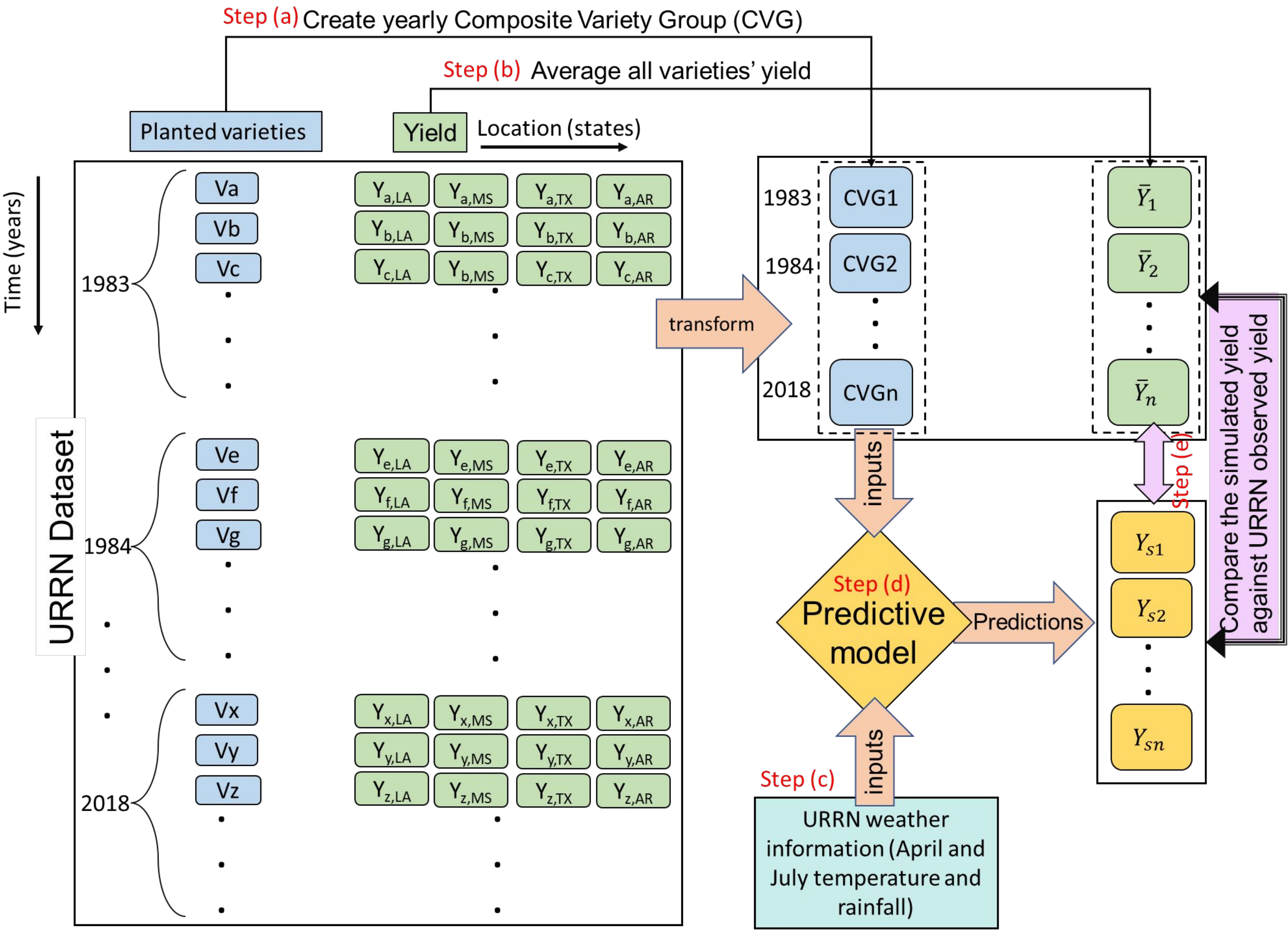

Figure S5. Framework of ensemble model evaluation against the URRN dataset. (a) Genotyped varieties from the URRN are grouped per year to generate Composite Variety Groups (CVGs) to determine overall allele frequencies (a 'bag of alleles'). **(b)** Observed yields from those genotyped varieties from the URRN are averaged to generate a single location-by-year observation. The ensemble model takes in **(c)** URRN weather and **(d)** the 'bag of alleles' genetic information from the CVGs to generate predicted yields, which are **(e)** compared with observed averages.

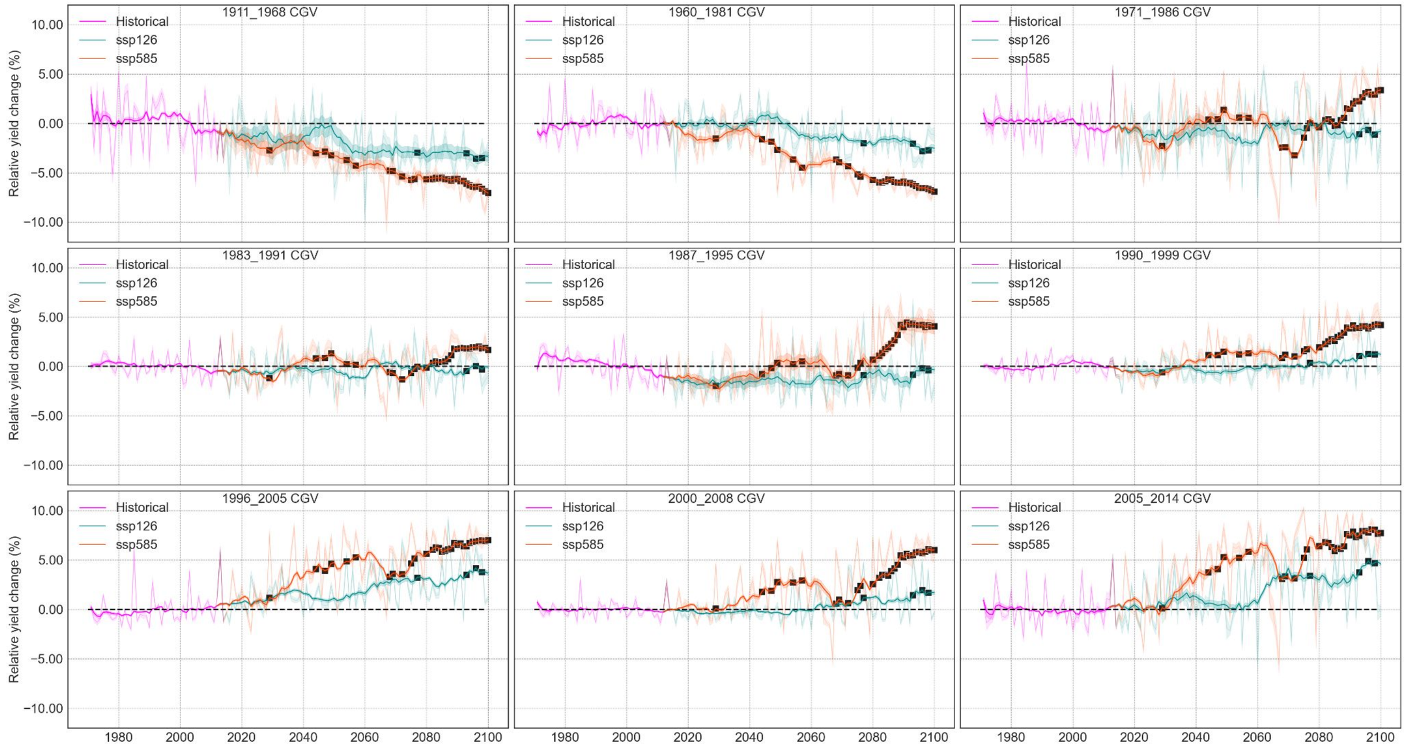

Figure S6. Sliding window analysis of future yield projections across Composite Variety Groups based on varietal year-of-release. Relative yield changes were computed using model-forecasted yields (2016-2100) relative to the average of model-backcasted yields (1970-2016) of the same CVG. The boxes on the line plots indicate the years with average maximum July temperature exceeding the threshold (40 C) considered for an an acceptable prediction and were removed from downstream analysis. Each CVG for the sliding window analysis was constructed using bins of 20 varieties with a step size of 10 varieties.

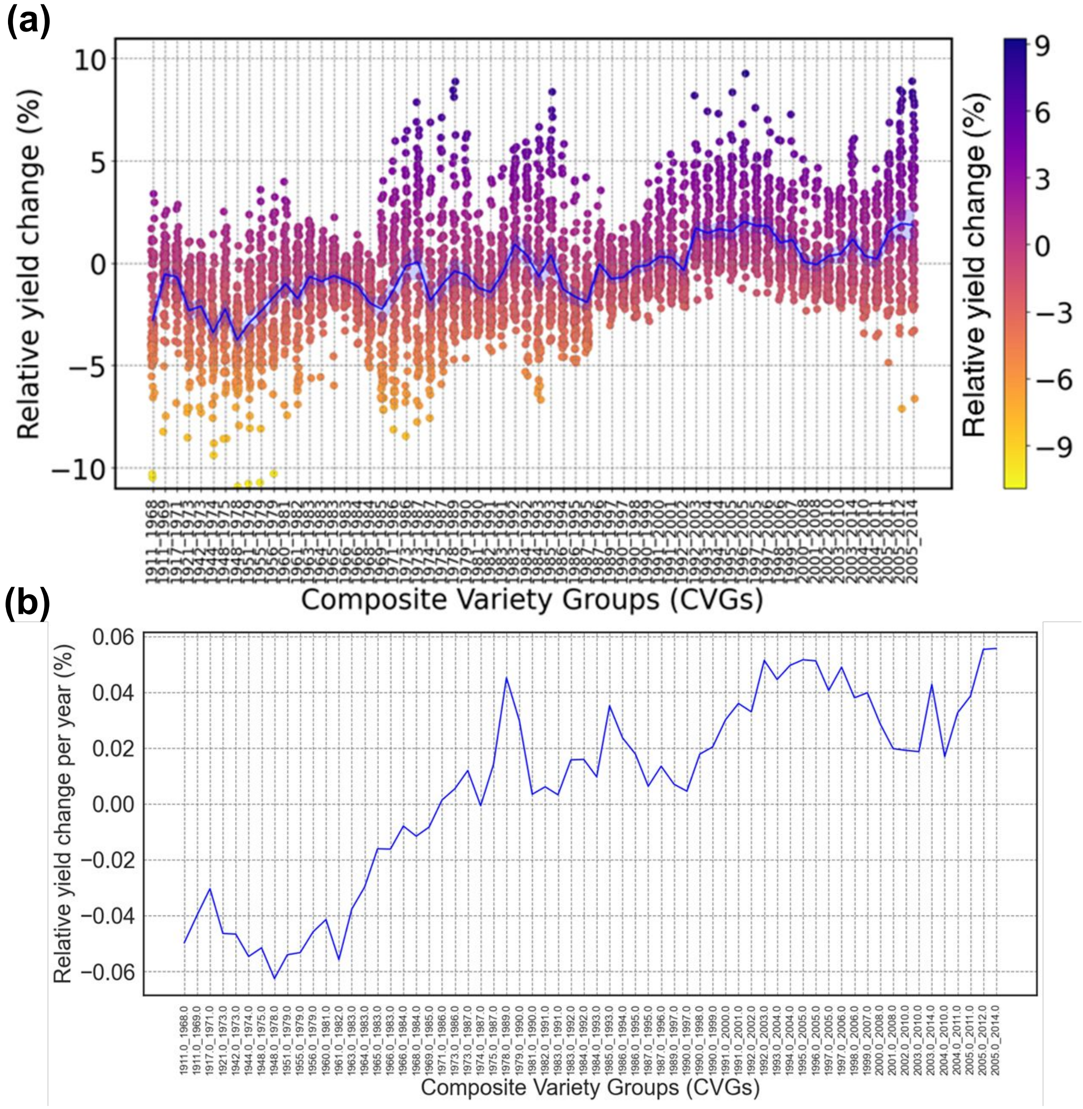

Figure S7. Forecasted yield performance of year-of-release Composite Variety Groups. (a) This plot depicts the same as Figure 4a, but with full x-axis labels indicating the range of year-of-release per Composite Variety Group. (b) Percentage of relative yield change per year across each CVG over its backcasted and forecasted simulated time series.

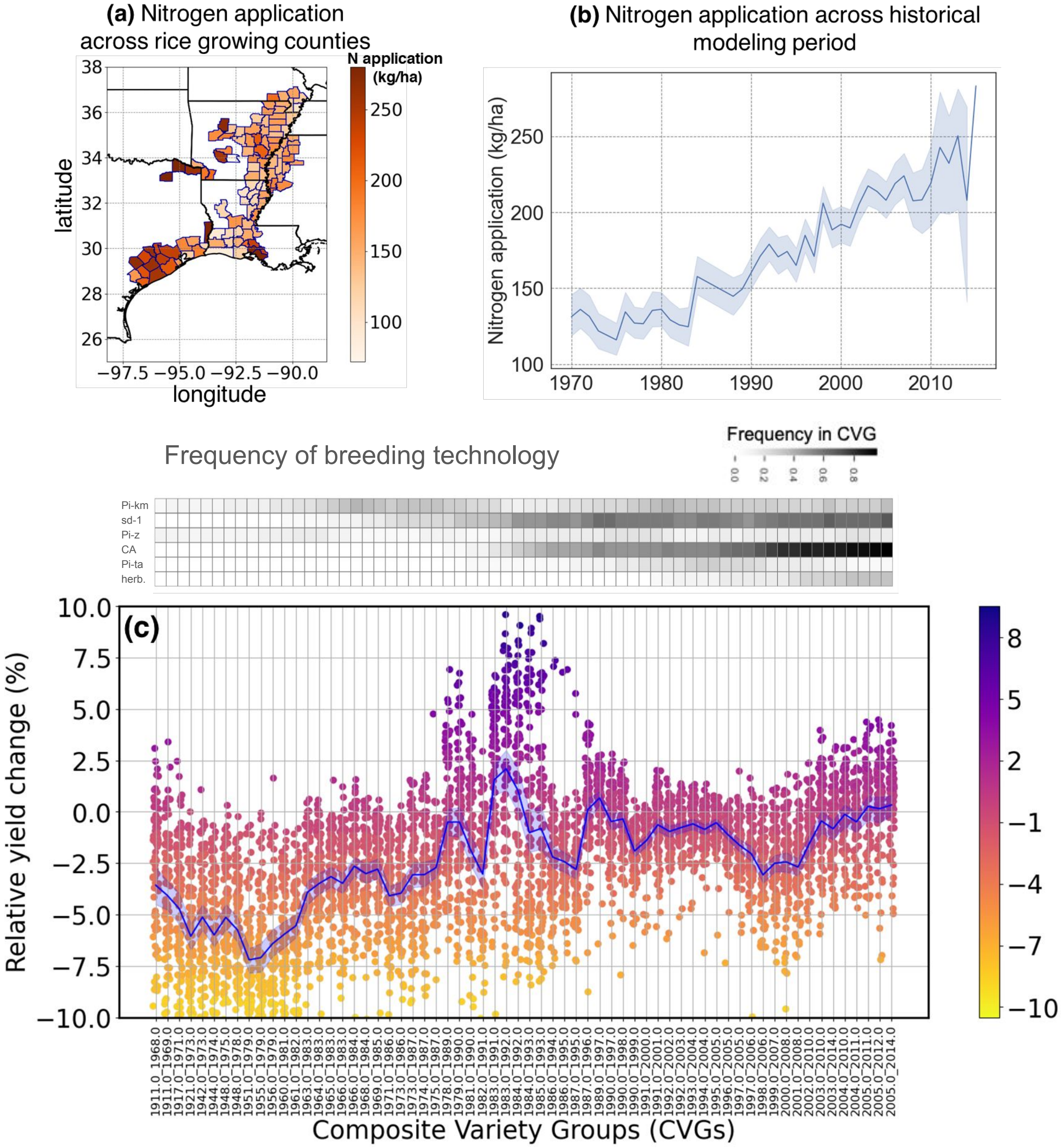

Figure S8. Historical nitrogen fertilizer application and its impact on relative yield change of Composite Variety Groups (CVGs). (a) Average historical county-level nitrogen application rate (kg/ha) for rice in the southern U.S. (b) Yearly nitrogen application rate (kg/ha) for rice in the southern U.S. computed as an average across rice growing counties. (c) Forecasted yield performance of year-of-release CVGs, considering the nitrogen impact within the Y=f(W, G, W*G, N, N*G) framework. Frequency of breeding trends in each CVG are shown as a heatmap above the main plot; these correspond to those shown in Figure 4a.

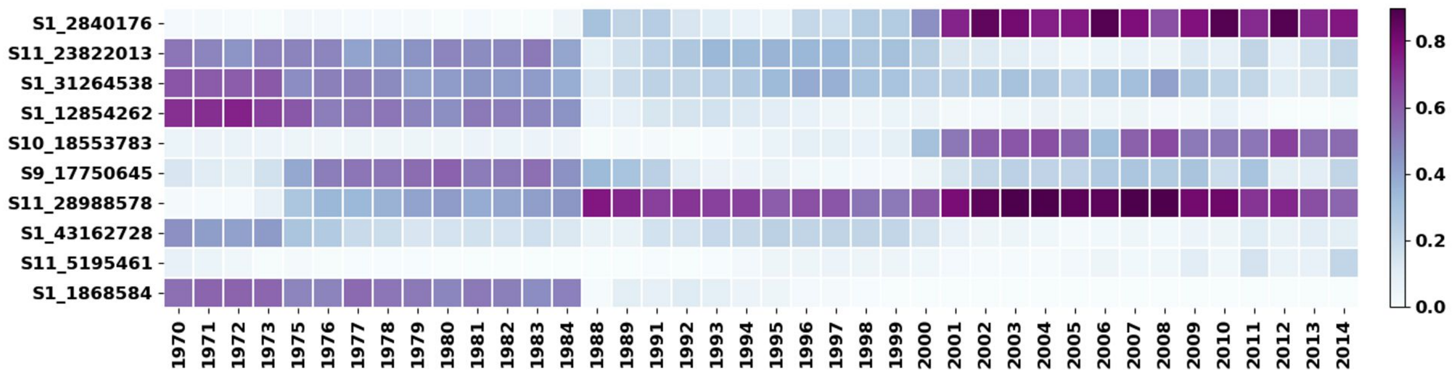

Figure S9. Examples of SNP allele patterns across time in the rice-growing region of study. Heatmap represents values of allele frequency across the entire region of 110 rice-growing counties of the minor allele at each SNP in each year. Each marker shown here is an example of groups of markers that have similar patterns.

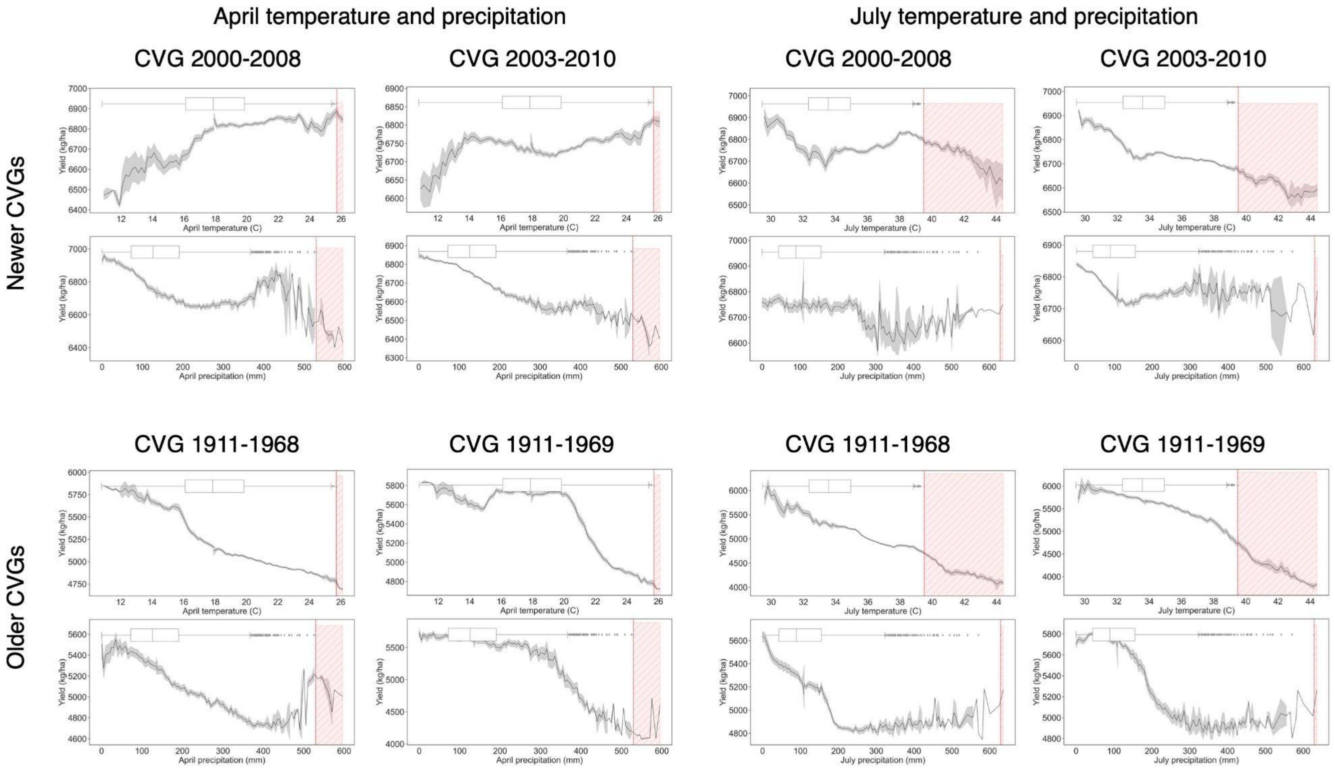

Figure S10. Examples of Composite Variety Group (CVG) responses to individual weather variables. The responses to April and July temperature and rainfall are illustrated for two historical CVGs (1911-1968 and 1911-1969) and two contemporary CVGs (2000-2008 and 2003-2010) for the SSP1-2.6 scenario. For each weather variable, the CVG's response was computed while keeping all other variables constant at their historical averages. In each plot, the shaded red portion represents the range of weather variable beyond the historical period (i.e., during future climate projections). The distribution of each weather variable is depicted in the upper section of each plot using a boxplot. The full set of CVG responses can be found in at Zenodo (https://doi.org/10.5281/zenodo.8346660).

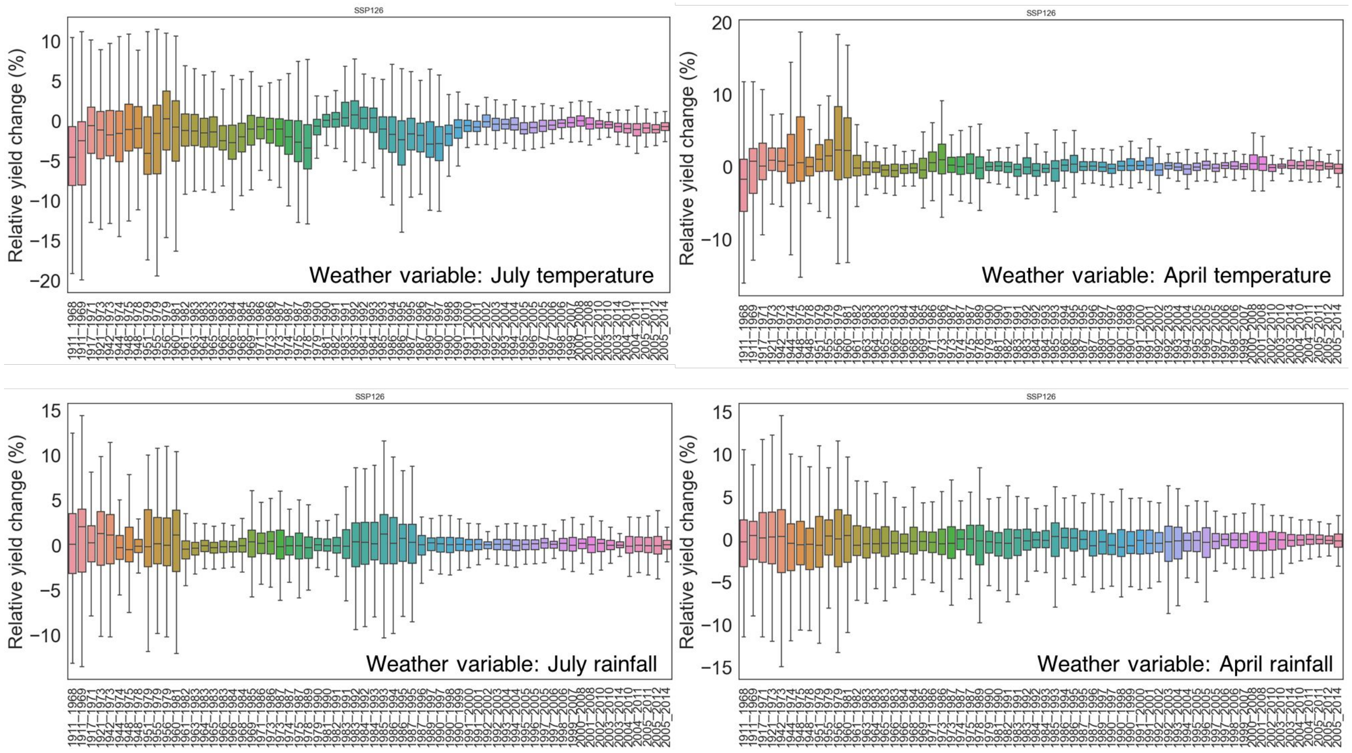

Figure S11. Summary of Composite Variety Group (CVG) responses to individual weather variables. The responses of each CVG (shown as forecasted yield relative to the historical yield) to April and July temperature and rainfall are illustrated using box plots. Each box plot represents a time series with illustrative examples provided in Figure S9. For each weather variable, the CVG's response is computed while keeping all other variables constant at their historical averages (SI Appendix supporting text).

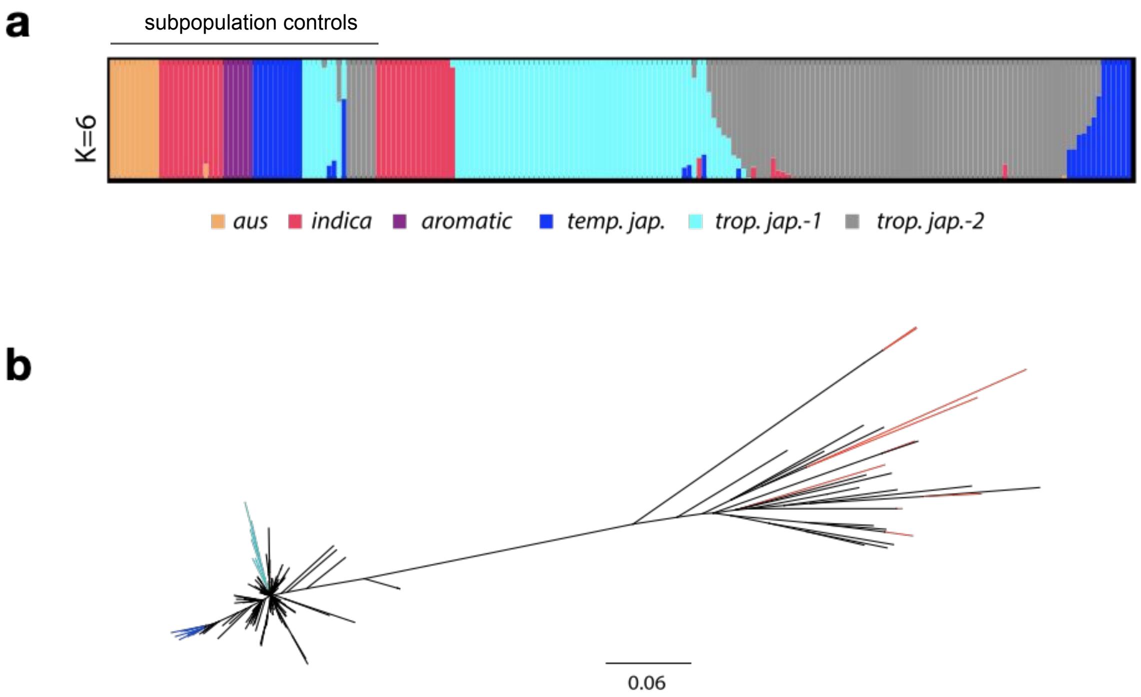

Figure S12. Population structure of U.S. rice 'Full Set'. 153 accessions (152 accessions genotyped as part of this study in addition to one accession (cv. Cypress) derived from previously published re-sequencing dataset of Duitama *et al.* [2015]) and 54 accessions of known subpopulation used as controls for the five subpopulations in *O. sativa*: *aus, indica, aromatic, temperate japonica,* and *tropical japonica*. Subpopulation controls are marked by the horizontal bar. **(a)** Results of fastStructure analysis at K=6, where all five subpopulations emerge. Two groups of *tropical japonica* (light blue and gray) are indicated by the analysis due to the large proportion of *tropical japonica* found in U.S. rice germplasm. 111500 SNP markers were used in this analysis. **(b)** Distance tree using a subset of markers with a minimum call rate of 90% (9004 markers). Colored lines indicate control accessions and black lines denote U.S. rice accessions. Colors for both panels of the figure are indicated by the legend in **(a)**.

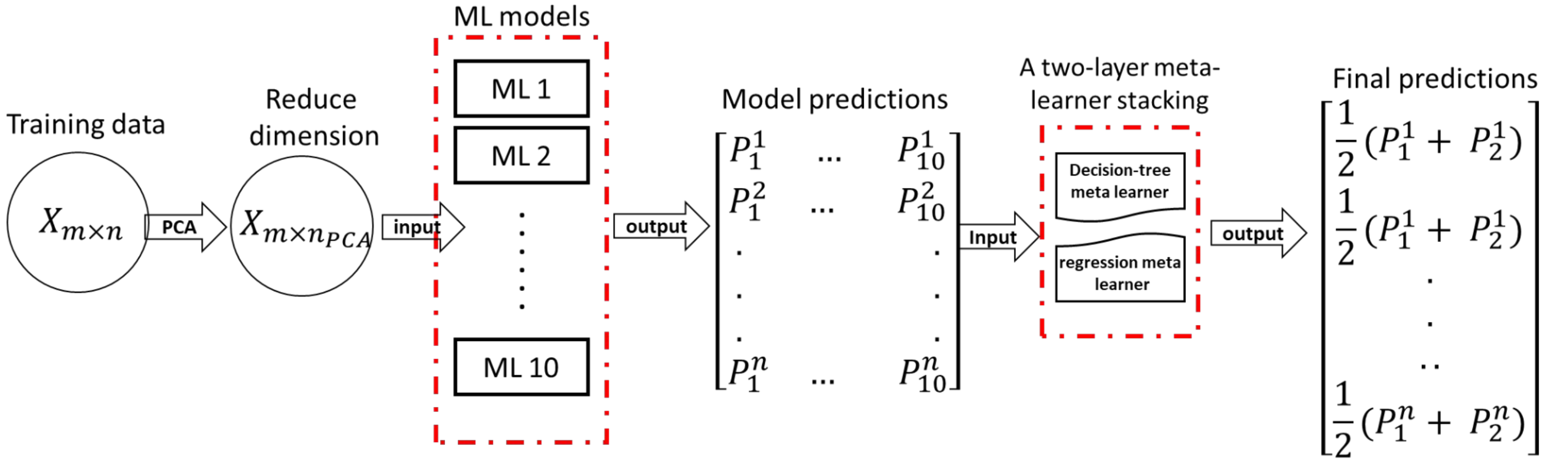

Figure S13. The ensemble modeling framework. Ten machine learning models (CatBoost, GradientBoost, RandomForest, AdaBoost, XGBoost, LASSO, Elastic net, Bayesian Ridge, Support vector, and Stochastic Gradient Descent) were hypertuned, trained, and tested using production data. These models were then stacked using a two-layer meta learner using the XGBoost and Lasso regressors, and their averaged outcome was considered for comparison with observed yields.

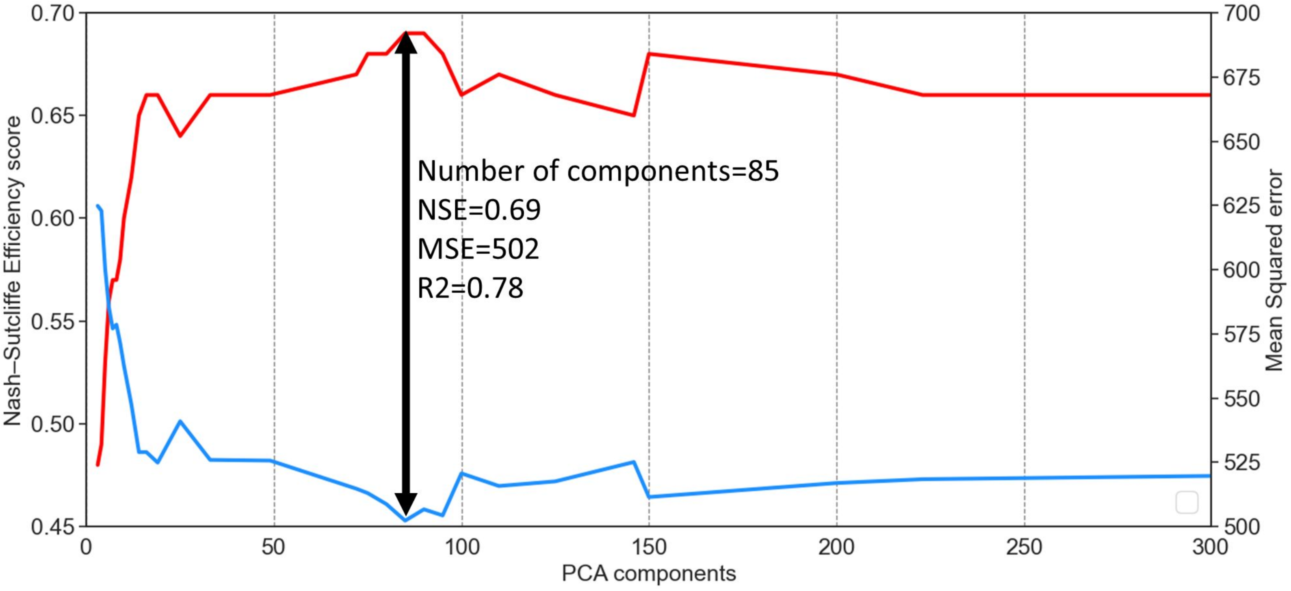

Figure S14. Principal component selection. Principal components analysis was used to reduce dimensionality of the combined genetic, weather, and genetic-weather interactions dataset for modeling. The accuracy of the model outcomes (average of the ten individual models) as a function of the number of principal components used for modeling. The red line shows the Nash–Sutcliffe Efficiency score (left y-axis) and the blue line shows the mean squared error (right y-axis).

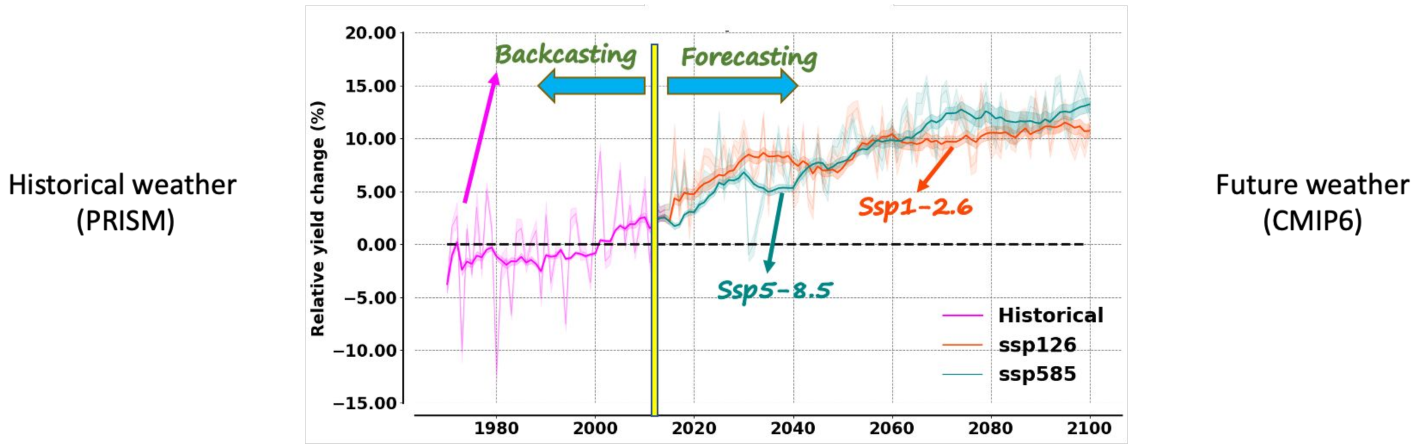

Figure S15. Explanation of backcasting and forecasting. An example of a backcasted and forecasted CVG. Backcasted predictions (using historical weather) are shown in magenta while forecasted predictions (using future weather) are shown in orange and blue, with colors indicating the Shared Socioeconomic Pathway (SSP). Relative yield change is computed based on dividing by the average of all backcasted predictions multiplied by 100%.
